# Supplementary material for: Smooth Interpolating Curves with Local Control and Monotone Alternating Curvature
Source: Comput Graph Forum. 2022 Oct 6;41(5):25–38. doi: 10.1111/cgf.14600 (PMC9827861; doi:10.1111/cgf.14600)
Supplement: Supplementary file 1 — Supplement Material [file CGF-41-25-s001.zip › Local-Smooth-Interpolating-MonoCurvature/extern/clothoids/submodules/quarticRootsFlocke/docs/readme.html]

Quartic Roots Solver — Quartic Roots v1.0.0

### Navigation

- index
- toc
- next
- previous
- Quartic Roots »
- Quartic Roots Solver

# Quartic Roots Solver¶

## Polynomial Roots Solver¶

Port to C++ of the algorithm of Norbert Flocke for polynomial roots up
to degree 4. Also Jenkins-Traub real polynomial root finder is ported
but is experimental for the moment.

## Usage¶

```
#include "PolynomialRoots.hh"

// ....

double coeffs[] = { 8, -8, 16,-16, 8,-8 }; // polynomial coeffs

double zeror[5], zeroi[5];
int    info[5];
int    degree = 5;

int ok = PolynomialRoots::roots( coeffs, degree, zeror, zeroi ); // ok < 0 failed
cout << " ok = " << ok << '\n' ;
for ( int i = 0 ; i < degree ; ++i )
  cout << zeror[i] << " + I* " << zeroi[i] << '\n';
```

To solve quadratic, cubic or quartic use specialized classes

```
Quadratic qsolve(a,b,c);
qsolve.info(cout);

Cubic csolve(a,b,c,d);
csolve.info(cout);

Quartic q4(a,b,c,d,e);
q4.info(cout);
```

look at the class definition to see how to access the computed roots.

## Repository and Online Documentation¶

- https://github.com/ebertolazzi/quarticRootsFlocke
- http://ebertolazzi.github.io/quarticRootsFlocke

## References¶

- **N.Flocke**

  Algorithm 954: An Accurate and Efficient Cubic and Quartic

  Equation Solver for Physical Applications

  ACM TOMS, vol 41, n.4, 2015
- **M.A. Jenkins and J.F.Traub**

  A Three-Stage Algorithm for Real Polynomials Using Quadratic
  Iteration

  SIAM Journal on Numerical Analysis

  Vol. 7, No. 4 (Dec., 1970), pp. 545-566

## Author¶

> Enrico Bertolazzi
>
> Dipartimento di Ingegneria Industriale
>
> Universita` degli Studi di Trento
>
> email:

### Quick search

### Table of Contents

- Quartic Roots Solver
  - Polynomial Roots Solver
  - Usage
  - Repository and Online Documentation
  - References
  - Author
- C++ API

«
hide menu

menu
sidebar
»

### Navigation

- index
- toc
- next
- previous
- Quartic Roots »
- Quartic Roots Solver

© Copyright 2021, Enrico Bertolazzi.
Created using Sphinx 4.2.0.
